# Intellectual disability and COVID-19: A bibliometric review

**DOI:** 10.3389/fpsyt.2022.1052929

**Published:** 2022-11-09

**Authors:** Jiangbo Ying, Giles Ming Yee Tan, Melvyn Weibin Zhang

**Affiliations:** ^1^East Region, Institute of Mental Health, Singapore, Singapore; ^2^Central Region, Institute of Mental Health, Singapore, Singapore

**Keywords:** intellectual disability, COVID-19, bibliometric analysis, scientific collaboration, research trends

## Abstract

**Background:**

During the COVID-19 pandemic, the vulnerabilities of certain groups of people have been highlighted, such as people with intellectual disability (ID). Although related research on ID has developed rapidly during the COVID-19 pandemic, the quantitative analysis of those research results has not been systematically performed through bibliometric analysis. Bibliometric analysis is a useful and rigorous method to explore large volumes of research data, and it allows researchers to extract quantitative information on distribution by author, time, country, and journal.

**Aim:**

The aim of the present study is to comprehensively analyze the current status and developing trends in publications on ID research related to and conducted during the COVID-19 pandemic.

**Methods:**

A bibliometric analysis was performed using the Web of Science database. Biblioshiny software was used to analyze and visualize the following information: main information of dataset, annual scientific production, journals which published the most relevant sources, most-cited authors, most-cited countries, most-cited global documents, word-cloud of keywords authors have used, and both the co-occurrence and co-citation networks.

**Results:**

A total of 450 publications were included. The average number of citations per document was 5.104. Among the top three journals, Journal of Applied Research in Intellectual Disabilities published 32 articles, Journal of Intellectual Disability Research published 29 articles, and British Journal of Learning Disabilities published 17 articles. The article with the title COVID-19 and People with Intellectual Disability: Impact of a Pandemic was the most cited with total 144 citations The United Kingdom had the most publications and had strong cooperative relationships with the United States, Canada, and Australia. The most popular keywords included mental health, autism, developmental disability, and lockdown. Thematic map analysis identified several possible clusters, including telemedicine, physical activities, and mental health.

**Conclusion:**

The present study provides a better understanding in this research field and may help clinicians, researchers and stakeholders to obtain more comprehensive view of ID and COVID-19. The insights gained from this analysis could inform future research.

## Introduction

The coronavirus disease 2019 (COVID-19), caused by the novel severe acute respiratory syndrome coronavirus 2 (SARS-CoV-2) (1), has led to global health crisis. On the 11th of March 2022, the World Health Organization declared COVID-19 a pandemic. The COVID-19 pandemic not only has posed a high risk to physical health, but also has robustly affected mental health (2). Research has indicated that people affected by COVID-19 have a high burden of mental health issues, such as sleep disorders, anxiety disorders, emotional disturbance and suicidal behavior (3). The level of mental health burden varies depending on many factors, such as the population groups, the stage of the pandemic, and socio-economic conditions (4). During the pandemic, the vulnerabilities of certain groups of people have been highlighted, such as the pregnancy women (5), the homeless (6), those with mental disorders (7), and people with intellectual disability (ID) (8).

People with ID have an increased risk of COVID-19 diagnosis, morbidity, and mortality (9, 10). The risk factors of COVID-19 morbidity and mortality among people with ID include increased age, living in a congregate setting, chronic kidney disease, heart disease and Down syndrome (11, 12). People with ID face isolation and precaution-related challenges, including emotional and behavioral disturbance from restricting usual activities and social isolation (8, 13). They also have other difficulties, such as loss of certain mental health services and community supports (14, 15). Globally, numerous studies have assessed the impacts of COVID-19 pandemic on the mental health conditions of people with ID in numerous countries, including United States (16), United Kingdom (17), Netherlands (18), Israel (19), Spain (20), and South Korea (21). Overall, people with ID are especially vulnerable to the psychosocial effects of the COVID-19 pandemic.

Although there is reasonable amount of research on ID during the COVID-19 pandemic, the quantitative analysis of ID research results related to COVID-19, to our knowledge, has not been systematically performed through bibliometric analysis. Bibliometric analysis is a useful and rigorous method for scientists to explore large volumes of research data, and it allows researchers to extract quantitative information on distribution by author, time, country and journal (22). This bibliometric approach has been used by researchers for various reasons, such as to assess the intellectual structure of a specific domain in the literature, to look for the emerging research trends (23) and hotspots in certain topics (24), and to uncover journal performance and research constituents (25). Bibliometric analysis has gained popularity in a variety of hot fields, such as interpretive structural modeling (26), Internet of Things (27), fuzzy theory (28), applied intelligence (29), sustainable tourism (30), and rough sets research (31). It has also been used for many physical and chronic medical conditions, such as obesity (32), cancer (24), and diabetes (33). It helps researchers better understand the direction of research in the field and identify key gaps to facilitate future research.

The aim of the present study is to comprehensively analyze the current status and developing trends in publications on ID research related to and conducted during the COVID-19 pandemic. Our focus is to: (1) assess the research outputs in publications; (2) construct global scientific collaboration networks among countries; (3) find the core countries, researchers and journals; and (4) explore the key topics and hotspots to guide future research and applications. The study has organized a comprehensive bibliometric analysis with the help of available software discussed in the upcoming subsection. The information synthesized will be of importance in the future conceptualization of new research involving individuals with ID.

## Materials and methods

For the purposes of this current bibliometric review, Web of Science (WOS) database was used for the identification of relevant articles. The WOS database was chosen, as it had been deemed superior as compared to Scopus or MEDLINE/PubMed for the following reasons. The WOS database allows for the extraction of many articles with full information, including the titles, author names, total citations and total download times, and it covers the citations of scientific publications since 1900 and has included all the high impact scientific journals (34, 35). It includes world-class indexes, including Social Sciences Citation Index and Science Citation Index Expanded. This database has two major strengths which are reference tracing and citation reporting, as it enables search within leading academic journals and citation networks and can powerfully trace references and citation activities to explore research outputs in a specific area (36). The WOS database has been used by researchers in bibliometric analyses in many areas, such as oral carcinoma (24), trigeminal neuralgia (37), and gut microbiota (38). For the identification of the relevant articles, we consulted an information specialist from the Lee Kong Chian School of Medicine, Nanyang Technological University, Singapore. The information specialist helped in the refinement of the search terminologies and strategies, to ensure that all the relevant articles were captured. The following search strategy was utilized for the identification of relevant articles: TS = (“COVID-19” or “COVID19” or “COVID 19” or “SARS-nCoV-2” or “SARS-CoV-2” or “2019-nCoV” or “novel coronavirus” or “coronavirus disease 2019” or “coronavirus disease-19” or “SARS coronavirus infection” or “severe acute respiratory syndrome coronavirus 2 infection”) AND TS = (“intellectual disabilit^*^” or “intellectual impairment” or “learning disabilit^*^” or “intellectual developmental disabilit^*^” or “mental retardation” or “learning difficult^*^” or “mental deficienc^*^” or “intellectual deficienc^*^” or “mental deficienc^*^” or “idioc^*^” or “intellectual development disorder^*^” or “intellectual development deficienc^*^”). The terminology TS refers to a search based on the topic of interest.

Using this approach, a total of 489 publications were retrieved from WOS, from the cross-sectional search that was conducted on 16 October 2022. No restrictions were implemented with regards to the WOS categories and the types of articles we sampled. Two independent researchers (MWZ and JY) screened the publications, for the identification of relevant articles. Articles were considered to be included if (a) their research ideas were related to ID and (b) one of their focuses was COVID-19. Articles were excluded if they did not mention people with learning difficulties, but focused on difficulties of learning methods, such as remote learning difficulties.

### Data analysis

The bibliometric data analysis was performed using Biblioshiny (RStudio, Version 1.4) (39). The software was used to analyze and visualize the following information: main information of dataset, annual scientific production, journals which published the most relevant sources, most cited authors, most cited countries, most cited global documents, word-cloud of keywords authors have used, and both the co-occurrence and co-citation networks.

## Results

### Analysis of publication outputs

**Figure 1:**
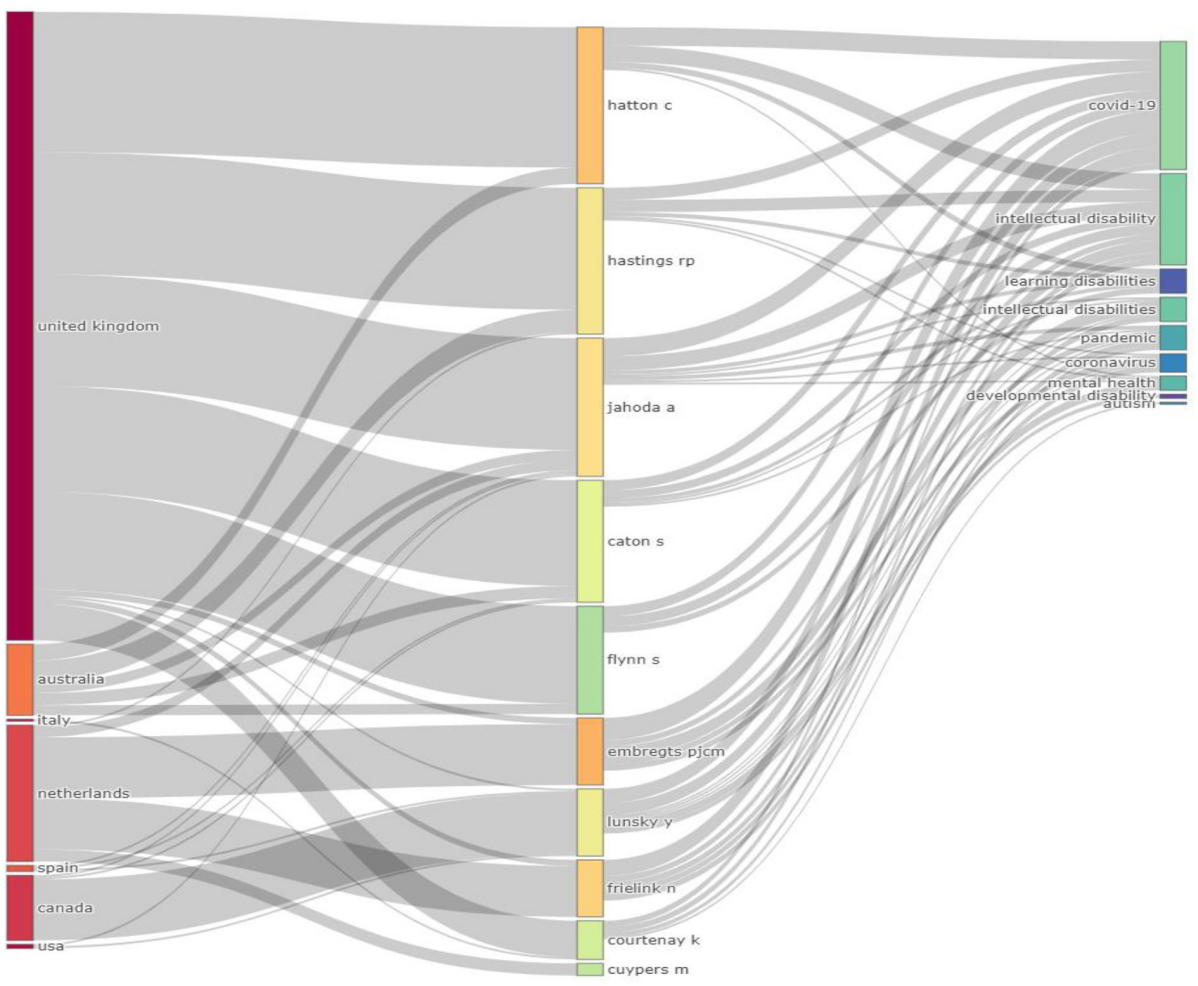
Three-field plot showing the association among countries, authors, and keywords. The left field shows the countries, the middle field the authors and the right field the authors' keywords.

A total of 450 publications were eventually included. The average number of citations per document was 5.104. Of these 450 publications, 290 were articles, 62 were early access articles, one was book review, 13 were editorials, one was early access editorial, 12 were letters, 34 were meeting abstracts, three was news items, eight were proceeding papers, 24 were reviews, and two were early access reviews. With regards to the annual scientific production, 54 were published in 2020, 204 in 2021 and 127 in 2022. Figure 1 presents the three-field plot of top authors, keywords used in the indexing of the articles and the countries where the authors originate. Figure 1 was generated with number of times limited to 10 per field. The left field shows the countries, the middle field the authors and the right field the authors' keywords. As shown in Figure 1, most authors are from the United Kingdom, and some of the keywords are pandemic, mental health, developmental disability and autism. For example, one of the authors, Hatton C, has keywords of COVID-19, Intellectual Disability, Learning disabilities and Mental Health. This figure helps us in understand the association between the keywords used, top-most authors involved in these publications and their corresponding countries of origin.

### Analysis of journals

The following tables illustrate the outcomes of the analysis pertaining to the journals in which articles were published and the leading authors in the field. Table 1 provides further information about the top 20 most productive journals and the corresponding number of articles published in that journal. Among the top three journals, Journal of Applied Research in Intellectual Disabilities published 32 articles, Journal of Intellectual Disability Research published 29 articles, and British Journal of Learning Disabilities published 17 articles. Thirteen out of the 20 journals were journals related to the sub-specialty of learning and intellectual disabilities.

**Table 1:** Journals in which articles were published.

**Journals**	**Articles**
Journal of Applied Research in Intellectual Disabilities	32
Journal of Intellectual Disability Research	29
British Journal of Learning Disabilities	17
Journal of Policy and Practice in Intellectual Disabilities	14
Advances in Mental Health and Intellectual Disabilities	10
International Journal of Developmental Disabilities	10
International Journal of Environmental Research and Public Health	10
BJPsych Open	9
Journal of Intellectual Disabilities	9
Tizard Learning Disability Review	8
Disability and Health Journal	7
Frontiers in Psychiatry	7
BMJ-British Medical Journal	6
Journal of Intellectual and Developmental Disability	6
Epilepsy and Behavior	5
Advances in Neurodevelopmental Disorders	4
Frontiers in Psychology	4
International Social Work	4
Journal of Autism and Developmental Disorders	4
Research in Developmental Disabilities	4

Table 2 reports an overview of the top 20 leading authors in the field and the corresponding number of articles that they have published. Embregts P and Hatton C ranked first with 12 articles, respectively.

**Table 2:** Leading authors in the field.

**Authors**	**Articles**
Embregts P	12
Hatton C	12
Frielink N	10
Jahoda A	10
Hastings RP	9
Lunsky Y	8
Caton S	7
Courtenay K	7
Cuypers M	6
Flynn S	6
Koks-Leensen M	6
Naaldenberg J	6
Taggart L	6
Bakker-Van Gijssel E	5
Beail N	5
Beyer S	5
Cooper V	5
Gillooly A	5
Heslop P	5
Langdon PE	5

Table 3 shows an overview of the analysis of the most globally cited publications. This information is pertinent for us in understanding which articles have had the most impact in the field. The article with the title COVID-19 and People with Intellectual Disability: Impact of a Pandemic was the most cited with total 144 citations. The second most cited was Intellectual and Developmental Disability and COVID-19 Case-Fatality Trends: TriNetX Analysis.

**Table 3:** Most globally cited publications.

**Author, year, journal**	**Publications**	**Total citations**	**Citations per year**
Courtenay K, 2020, Irish J Psychol Med	COVID-19 and people with intellectual disability: impacts of a pandemic	144	48.00
Turk MA, 2020, Disabil Health J	Intellectual and developmental disability and COVID-19 case-fatality trends: TriNetX analysis	141	47.00
Willner P, 2020, J Appl Res Intellect	Effect of the COVID-19 pandemic on the mental health of carers of people with intellectual disabilities	87	29.00
Jeste S, 2020, J Intell Disabil Res	Changes in access to educational and healthcare services for individuals with intellectual and developmental disabilities during COVID-19 restrictions	84	28.00
Landes Sd, 2020, Disabil Health J	COVID-19 outcomes among people with intellectual and developmental disability living in residential group homes in New York State	84	28.00
Becker SP, 2020, J Adolescent Health	Remote learning during COVID-19: examining school practices, service continuation, and difficulties for adolescents with and without attention-deficit/hyperactivity disorder	74	24.67
Vai B, 2021, Lancet Psychiat	Mental disorders and risk of COVID-19-related mortality, hospitalization, and intensive care unit admission: a systematic review and meta-analysis	64	32.00
Embregts P, 2022, Int J Dev Disabil	A thematic analysis into the experiences of people with a mild intellectual disability during the COVID-19 lockdown period	50	50.00
Smith K, 2020, Jmir Ment Health	COVID-19 and telepsychiatry: development of evidence-based guidance for clinicians	50	16.67
Semenzato L, 2021, Lancet Reg Health-Eu	Chronic diseases, health conditions and risk of COVID-19-related hospitalization and in-hospital mortality during the first wave of the epidemic in France: a cohort study of 66 million people	48	24.00
Theis N, 2021, Disabil Health J	The effects of COVID-19 restrictions on physical activity and mental health of children and young adults with physical and/or intellectual disabilities	44	22.00
Courtenay K, 2020, Bmj-Brit Med J	Covid-19: challenges for people with intellectual disability	39	13.00
Zaagsma M, 2020, J Intell Disabil Res	The use of online support by people with intellectual disabilities living independently during COVID-19	37	12.33
Duan WJ, 2020, Soc Sci Med	COVID-19-related stigma profiles and risk factors among people who are at high risk of contagion	37	12.33
Alexander R, 2020, J Policy Pract Intel	Guidance for the treatment and management of COVID-19 among people with intellectual disabilities	36	12.00
Tromans S, 2020, BJPsych Open-A	Patterns of use of secondary mental health services before and during COVID-19 lockdown: observational study	36	12.00
Williamson EJ, 2021, BMJ-Brit Med J	Risks of COVID-19 hospital admission and death for people with learning disability: population based cohort study using the OpenSAFELY platform	30	15.00
Bailey T, 2021, J Intell Disabil Res	COVID-19 impact on psychological outcomes of parents, siblings and children with intellectual disability: longitudinal before and during lockdown design	29	14.50
Embregts P, 2021, J Appl Res Intellect	Experiences and needs of direct support staff working with people with intellectual disabilities during the COVID-19 pandemic: a thematic analysis	24	12.00
Amor AM, 2021, J Intell Disabil Res	Perceptions of people with intellectual and developmental disabilities about COVID-19 in Spain: a cross-sectional study	24	12.00

### Analysis of scientific collaboration

**Figure 2:**
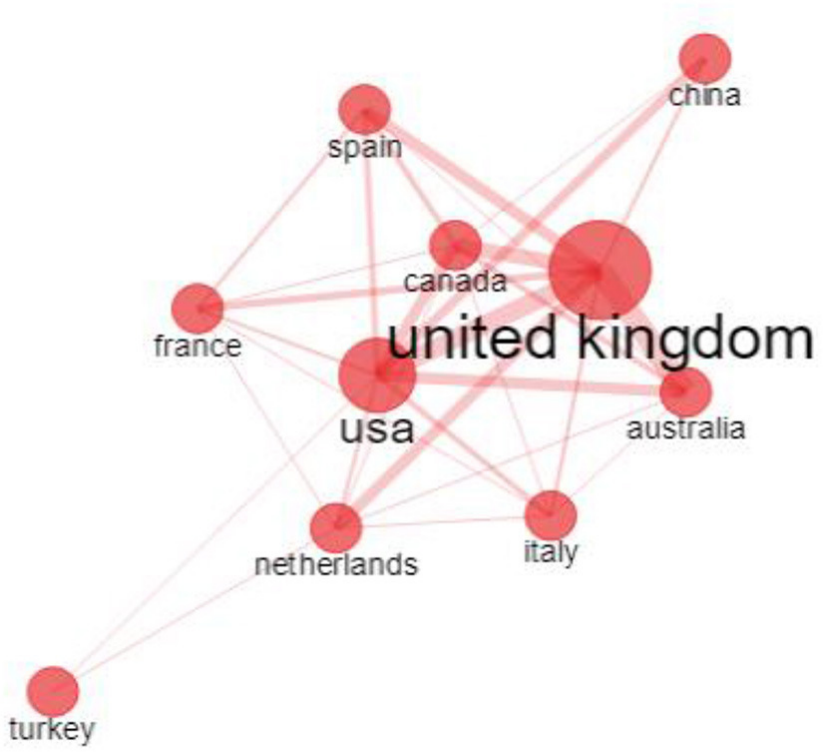
Visualization map of the scientific collaboration among countries. The nodes and lines between them show the countries and their cooperative relationships, respectively. The size of the node indicates the number of publications, and the width of the line represents the strength of the relationship.

The collaboration network map among countries is presented in Figure 2. Figure 2 was generated with the number of countries limited to 10. The nodes and lines between them show the countries and their cooperative relationships, respectively. The size of the node indicates the number of publications, and the width of the line represents the strength of the relationship. The United Kingdom had the most publications and had strong cooperative relationships with the United States, Canada, and Australia.

### Analysis of keywords

**Figure 3:**
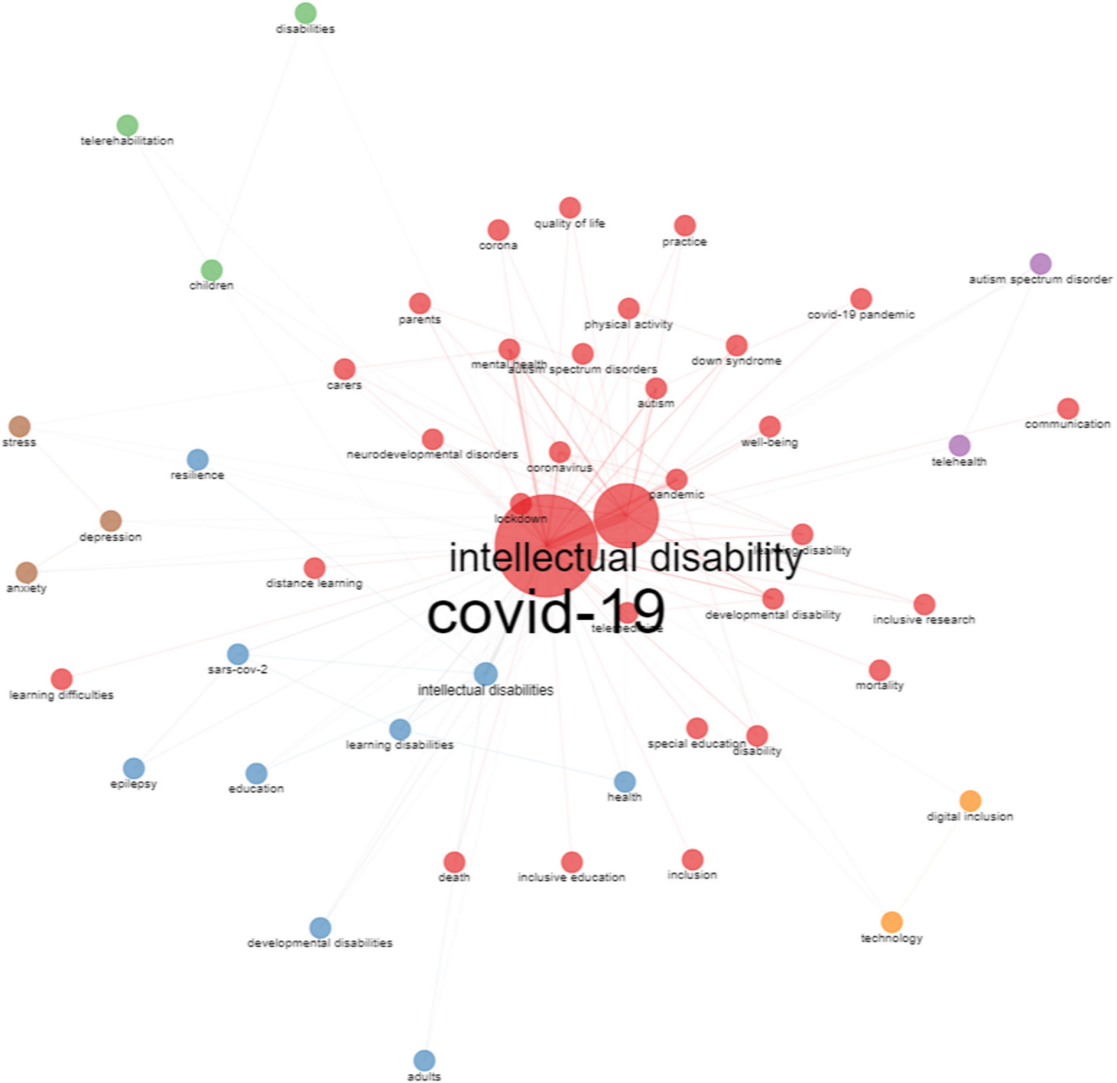
An overview of the co-occurrence of keywords. The nodes and linking lines between them represent the keywords and their co-occurrence relationships, respectively.

Keywords are essential in identifying the research trends. The most popular words were COVID-19 (*n* = 238), intellectual disability (*n* = 123), intellectual disabilities (*n* = 40), mental health (*n* = 33), pandemic (*n* = 29), autism (*n* = 23), coronavirus (*n* = 23), lockdown (*n* = 20), learning disabilities (*n* = 16), developmental disability (*n* = 15), learning disability (*n* = 15), COVID-19 pandemic (*n* = 13), and Down syndrome (*n* = 13). Figure 3 provides an overview of the co-occurrence of the authors' keywords. Figure 3 was generated with the number of keywords limited to 50. The nodes and linking lines between them represent the keywords and their co-occurrence relationships, respectively. For example, Down syndrome has co-occurrence with Physical Activity, Autism, COVID-19, COVID-19 Pandemic, and Intellectual Disability. The terminologies Intellectual Disability and COVID-19 are over-represented in the figure generated by Biblioshiny as they are the key terms used in the search strategy. They cannot be removed in view of the limitations of the software Biblioshiny with regards to figure preparation.

### Analysis of topics trends

**Figure 4:**
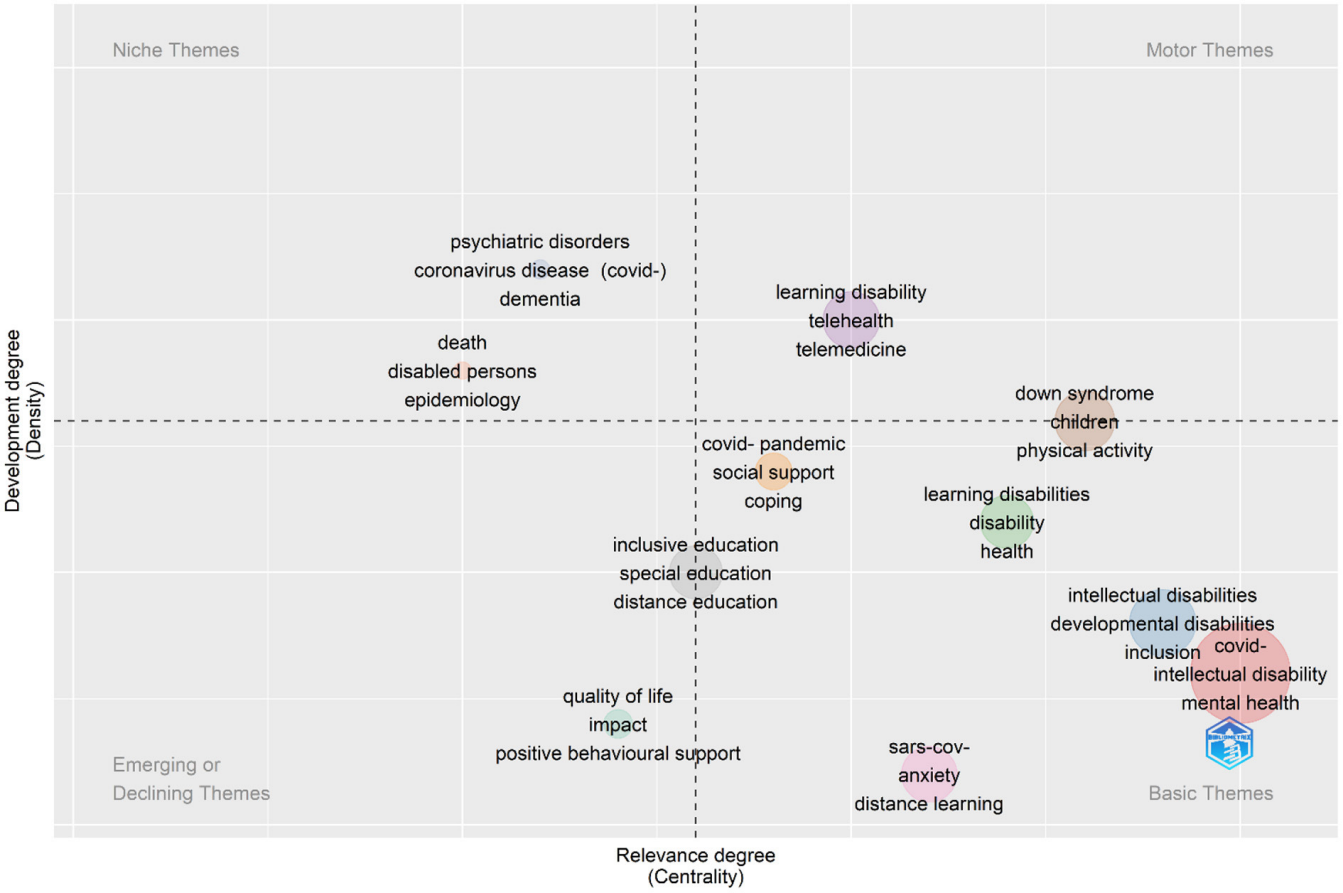
Thematic map analysis. Upper-right quadrant: motor themes—high density (developed) and high centrality (relevant); upper-left quadrant: niche themes—high density (developed) and low centrality (less relevant); lower-left quadrant: emerging or declining themes—low density (less developed) and low centrality (less relevant); lower-right quadrant: basic themes—low density (less developed) and high centrality (relevant).

Figure 4 provides the results of thematic map analysis. The thematic map was generated with the number of keywords limited to 250. A thematic map allows four typologies of themes to be defined based on the quadrant where they are placed. Themes in the upper-right quadrant are known as motor themes which are characterized by both high density and centrality, meaning they are developed and relevant for the research field. Themes in the upper-left quadrant are niche themes which are developed (high density) but isolated (low centrality). Themes in the lower-left quadrant are emerging or declining themes, as they have both low centrality and density indicating they are weakly developed and marginal. Themes in the lower-right quadrant are basic themes which are characterized by high centrality (relevant) and low density (less developed). As shown in Figure 4, there are a few relevant themes in this research field, including telemedicine, physical activities and social. The themes of intellectual disability and COVID-19 are over-represented in the figure generated by the software Biblioshiny as they are the key terms used in the search strategy. They were not removed as it could enable the visualization of the relationships between keywords.

## Discussion

This is the first bibliometric review that has explored all the publications relating to ID and COVID-19. The top journals which published most articles and the top articles with most citations were identified. The collaboration network among countries was described and important themes in the research area were explored. The analysis of the top journals and top globally cited articles helped in the identification of articles that were potentially of impact in the field. The collaboration networks, trending keywords and topic trends could be used as a reference for future research.

Journal analysis can provide useful information to help researchers to select appropriate journals for article submission. In the present study, it was found that only the top 2 journals, which were Journal of Applied Research in Intellectual Disabilities and Journal of Intellectual Disability Research, published more than 20 articles related to ID and COVID-19. It indicated a skewed distribution of the literature distribution across journals. In some research fields, researchers might have difficulty in choosing the most appropriate journals due to lack of knowledge or experience (38), but in research related to ID and COVID-19, it appeared most authors would aim to publish their articles in the top journals. This is likely because clinicians in the field of ID will need subspecialty training and are more aware of the top scientific journals. It is also heartening to note that not all articles specific to COVID-19 and intellectual disabilities are published in sub-specialty journals, but general journals like Frontiers in Psychiatry and BJPsych Bulletin also have considered these articles. It is important to highlight this as well, as this provides alternatives for researchers and academics to consider. Moreover, publications in these general journals also highlight that topics related to intellectual disability could reach out to a wider audience group. In addition, among the top 20 journals with highest publications, three journals (Frontiers in Psychiatry, Frontiers in Psychology, and Frontiers in Education) are from Frontiers. Frontiers has become a leading open access scholar publisher, and it ranks as the third most-cited publisher among the 20 largest publishers with an average of 4.8 citations per article (40).

The present study found that only two articles had more than 100 citations. These two articles were Turk's Intellectual and Developmental Disabilities and COVID-19 Case-Fatality Trends: TriNetX Analysis (41) and Courtenay's COVID-19 and People with Intellectual Disability (8). Turk's study highlighted that COVID-19 might present a greater risk to people with ID, especially at younger ages (41). Courtenay's review discussed the effects of COVID-19 pandemic on people with ID (8). The findings from these articles are of importance, given that the COVID-19 pandemic is still on-going, and it is important that fellow researchers consider the findings when conceptualizing their future research. Several other studies are also of significant, and have citations well above 50, including a study by Willner et al. (17) that examined the impact on the pandemic on caregivers' mental health, and a survey by Jeste et al. (16) that explored the access of individuals with intellectual disabilities to education and healthcare resources. The findings from these papers have important implications for policy planning pertaining to efforts to assist caregiving, and to further assist these individuals when education and healthcare access is restricted during the heights of the pandemic. The analysis of the top 20 articles also revealed that there has been qualitative research done in the field, such as a thematic analysis by Embergts et al. (42). Qualitative research could yield different insights into a particular subject matter, as compared to that of quantitative research.

Collaboration network analysis can provide important information for evaluating scientific collaborations and identifying key cooperators. Among countries, the analysis indicated that the United Kingdom and the United States had an advantage in this field, possibly because of their better economics and higher expenditure in scientific research. The findings in the present study were consistent with those in systematic reviews about ID research not related to the COVID-19 pandemic. In those systematic review publications, most articles were also from the United Kingdom or the United States (43, 44). For example, in a recent systematic review of the existing literature on complicated grief in people with ID, 18 relevant research articles were identified, but 10 of them were from the United Kingdom or the United States (43). The finding suggests that these two countries are well-developed in research related to ID and COVID-19. This information may be useful for researchers who would like to decide which country to choose for further training or collaboration.

According to the co-occurring keyword analysis, this study managed to identify some of the frequent hotspots in this field, including autism, Down syndrome and mental health. ID and autism frequently occur as comorbidities (45), and both of them may share many of the same susceptibility genes and variants (46). People with Down syndrome on average have mild to moderate intellectual disability (47). It has been reported that people with Down syndrome have increased prevalence of mental health issues (48). The prevalence of mental disorders is also higher among people with ID, and it has been reported that the rate of mental health problems in children and adolescents with ID is 3 to 4 times higher than those typically developing individuals (49, 50). Thus, the frequent co-occurring keywords could be explained by the common comorbidities among people with ID.

The thematic map analysis in the present study identified a few important themes that have been most studied, including physical activities, telemedicine and social support. Physical activities appear to have positive effects on mental health in children and adolescents with ID, but long term effects need to be assessed (51). Telemedicine can be an effective method of supporting the treatment and rehabilitation of people with ID, though there are risks (52). Social support is very important for people with ID. Social support is considered one of the import factors mediating the quality of life of people with ID and their family members (53). It has been reported that social support can enhance maternal wellbeing in women with ID (54). However, it has also been mentioned that more investigations are need to design and test the most effective social support (55). Overall, further research is needed to help people with ID in terms of physical activities, telemedicine, and social support.

Like with other bibliometric analysis, there are several limitation in this current study. First, the publications were extracted from the WOS database, which might have excluded some studies from other databases, such as Scopus. The reasons as to why the usage of WOS was chosen have been explicitly mentioned in the methodology. Second, the database was searched at a specific time point, and there might be articles published more recently that had been missed. Third, self-citations might have influenced the results of the citation analysis. Despite these limitations, this descriptive bibliometric study could contribute new information about ID research during the COVID-19 pandemic and reflect the overall state of and general trends in this research filed.

## Conclusion

The present study, to our knowledge, is the first bibliometric analysis to explore the publications relating to ID and COVID-19. It provides a better understanding in this research field and may help clinicians, researchers and stakeholders to obtain more comprehensive view of ID and COVID-19. The insights gained from this analysis could inform future research.

## Data availability statement

The datasets presented in this study can be found in online repositories. The names of the repository/repositories and accession number(s) can be found in the article/Supplementary material.

## Author contributions

JY, GT, and MZ designed the study. JY and MZ acquired the data and performed statistical analyses. JY drafted the manuscript. GT and MZ reviewed and edited the manuscript. All authors critically revised the article and approved the final version of the manuscript.

## Conflict of interest

The authors declare that the research was conducted in the absence of any commercial or financial relationships that could be construed as a potential conflict of interest.

## Publisher's note

All claims expressed in this article are solely those of the authors and do not necessarily represent those of their affiliated organizations, or those of the publisher, the editors and the reviewers. Any product that may be evaluated in this article, or claim that may be made by its manufacturer, is not guaranteed or endorsed by the publisher.
